# Supplementary material for: Impact of symmetry in local learning rules on predictive neural representations and generalization in spatial navigation
Source: PLoS Comput Biol. 2025 Jun 23;21(6):e1013056. doi: 10.1371/journal.pcbi.1013056 (PMC12184951; doi:10.1371/journal.pcbi.1013056)
Supplement: S1 Appendices — (PDF) [file pcbi.1013056.s001.pdf]

# Appendices for 'Impact of symmetry in local learning rules on predictive neural representations and generalization in spatial navigation'

Janis Keck[*], Caswell Barry[†], Christian F. Doeller [†], Jürgen Jost[†]

May 18, 2025

## A Learning Rule

In this appendix we are going to provide some additional insights into the learning rule we have employed. Furthermore, we will provide some informal derivation on the expected weights that should be learned under this learning rule. Recall hence our learning rule, which with parameters $\alpha = 1, \beta = 0$ entails the update of synaptic weights $W$ given pre- and postsynaptic activities $p_{pre}, p_{post}$ as

$$\Delta W = (p_{post} - W p_{pre}) p_{pre}^T. \tag{1}$$

This learning rule can be seen as performing a sort of conditional expectation objective: At convergence, the expected update in synaptic weights, given the current activity, should be zero

$$\mathbb{E}\left[\Delta W | p_{pre}\right] = 0. \tag{2}$$

We have that this holds in general only if

$$W p_{pre} = \mathbb{E}\left[p_{post} | p_{pre}\right]. \tag{3}$$

Note that $W$ will also appear on the right hand side, as $p_{post}$ will depend on $W$ - thus, this equation still has to be solved for these weights. Now in some sense, this learning rule is very similar in spirit to classical TD-learning, since also there one tries to fulfill a conditional expectation equation (the Bellman equation) and works towards a solution by bootstrapping [1]. Indeed, also in [2] this learning rule was derived starting from the assumption of TD-learning with function approximation.

*Corresponding author: janis.keck@maxplanckschools.de
†Shared senior authorship

Of course, the equation Equation 2 above will typically not have an exact solution, since conditioning on an input is in general a nonlinear operation. However, in special cases there will be a solution: If for example we have a conditional Gaussian relationship, that is $p_{post}|p_{pre} \sim \mathcal{N}(p_{post}; Ap_{pre}, \sigma)$, then an exact solution is possible: Then $\mathbb{E}[p_{post}|p_{pre}] = Ap_{pre}$ and hence $W = A$ solves the equation - this example serves only as an illustration of possible solutions and there is no biological meaning intended. In general, one can understand the learning rule as enforcing a simple form of a predictive coding objective, where the weights are adapted in such a way that postsynaptic activity becomes predictable from presynaptic activity [3]. Indeed, recall that the conditional expectation $\mathbb{E}\left[p_{post}|p_{pre}\right]$ is the best prediction of $p_{post}$, given $p_{pre}$. Thus, if Equation 2 is satisfied, $W$ indeed realizes this optimal prediction.

Let us now understand how the predictive coding rule learns successor representations.

## A.1 Learned representations through the predictive coding rule

Consider first a feedforward network with activities

$$p_{t+1} = \gamma V \phi_t + (1-\gamma)\psi_t. \tag{4}$$

Here, $\phi_t(s_t)$ is the input through the feedforward weights $V$ and $\psi_t(s_t)$ is external input, both depending on the state $s_t$ of a Markov process.

**Lemma 1.** *Applying the rule for the stationary weights from Equation 2 to the synaptic weights $V$ and pre- and postsynaptic activities $\phi_t, p_t$ respectively, yields the equation*

$$(1-\gamma)\mathbb{E}[\psi_{t+1}|s_t] = V(\phi_t - \gamma\mathbb{E}[\phi_{t+1}|s_t]) \tag{5}$$

*Proof.* In this situation, we have $p_{post} = \gamma V \phi_{t+1} + (1-\gamma)\psi_{t+1}, p_{pre} = \phi_t$. Thus, the equation that the weights have to fulfill at convergence reads

$$V\phi_t = \mathbb{E}[\gamma V \phi_{t+1} + (1-\gamma)\psi_{t+1}|\phi_t]. \tag{6}$$

Now as we assume an injective relationship of states $s_t$ and features, we may replace the conditioning on presynaptic activity by conditioning on hidden states $s_t$. Then pulling the weights $V$ out of the expectation due to linearity and then bringing all terms involving $V$ to one side yields the result. □

We assume the case of a finite state space, where we can write $\phi(s_t) = \Phi e_{s_t}, \psi(s_t) = \Psi e_{s_t}$, for matrices $\Phi, \Psi$ which we assume invertible. The equation is then

$$(1-\gamma)\Psi\mathbb{E}[e_{s_{t+1}}|s_t] = V\left(\Phi e_{s_t} - \gamma\Phi\mathbb{E}[e_{s_{t+1}}|s_t]\right) \tag{7}$$

It is then not hard to see that a solution is

$$V = (1-\gamma)\Psi P^T \left(\mathrm{Id} - \gamma P^T\right)^{-1} \Phi^{-1}. \tag{8}$$

But this is now just successor features (up to one timestep) in another basis: We have that applying $V$ to a feature vector $\phi_t$ yields

$$V\phi_t = (1-\gamma)\Psi P^T \left(\mathrm{Id} - \gamma P^T\right)^{-1} \Phi^{-1}\phi_t \tag{9}$$

$$= (1-\gamma)\Psi P^T \left(\mathrm{Id} - \gamma P^T\right)^{-1} e_{s_t} \tag{10}$$

$$= (1-\gamma)\sum_{k=1}^{\infty} \gamma^{k-1}\mathbb{E}\left[\psi_{t+k}|\phi_t\right] \tag{11}$$

which is hence indeed the successor representation of $\psi$ conditional on the features in $\phi$. In particular, plugging this solution for $V$ into equation Equation 4 then just includes the current timestep into the sum

$$p_t = (1-\gamma)\sum_{k=0}^{\infty} \gamma^{k}\mathbb{E}\left[\psi_{t+k}|\phi_t\right]. \tag{12}$$

The situation for the recurrent net is maybe even simpler (cf. [2]): Assume a network with activity

$$p_t = (1-\gamma)(\mathrm{Id} - \gamma W)^{-1}\phi_t. \tag{13}$$

Then Equation 2 for the weights $W$ becomes

$$(1-\gamma)(\mathrm{Id} - \gamma W)^{-1}\mathbb{E}[\phi_{t+1}|\phi_t] = W(1-\gamma)(\mathrm{Id} - \gamma W)^{-1}\phi_t. \tag{14}$$

Since $W$ and $(\mathrm{Id} - \gamma W)^{-1}$ commute, it is again not hard to see by similar algebraic manipulations as before, that a solution is

$$W = \Phi P^T \Phi^{-1}, \tag{15}$$

and hence when plugging $W$ back into the equation for $p$,

$$p_t = (1-\gamma)(\mathrm{Id} - \gamma \Phi P^T \Phi^{-1})^{-1}\phi_t = (1-\gamma)\sum_{k=0}^{\infty} \gamma^{k}\mathbb{E}\left[\phi_{t+k}|\phi_t\right]. \tag{16}$$

Thus we see that now $W$ encodes a successor representation for features $\phi$, conditioned on $\phi$ itself.

Finally, we want to ask what happens when the input to the feedforward-layer itself is also a successor representation, under the same prediction horizon $\gamma$. That is, when the matrix of input features is $\Phi = \tilde{\Phi}(\mathrm{Id} - \gamma P^T)^{-1}$, for some other set of features $\tilde{\Phi}$? In this case the matrix $V$ takes the particularly easy form $V = \Psi P^T \tilde{\Phi}^{-1}$, which is just the one step prediction of features $\Psi$ from features $\tilde{\Phi}$ (analogous to the recurrent case above). We see that still in this case $p_t$ will encode successor features, just conditional on the basis $\tilde{\Phi}$: Plugging in

the solution for $V$ into the equation for $p_t$ yields

$$\begin{aligned} p_t &= \gamma \Psi P^T \tilde{\Phi^{-1}} \phi_t + (1-\gamma)\psi_t && (17) \\ &= \gamma \Psi P^T \tilde{\Phi^{-1}} \tilde{\Phi} (\mathrm{Id} - \gamma P^T)^{-1} e_{s_t} + (1-\gamma)\psi_t && (18) \\ &= \gamma \Psi P^T (\mathrm{Id} - \gamma P^T)^{-1} e_{s_t} + (1-\gamma)\psi_t && (19) \\ &= (1-\gamma) \sum_{k=0}^{\infty} \gamma^k \mathbb{E}\left[\psi_{t+k} | \tilde{\phi}_t\right]. && (20) \end{aligned}$$

This shows that when one has a feedforward network which itself receives successor features as inputs, the result will again be successor features.

The limits derived in this section give the right intuition and are indeed the correct ones, we give a more formal analysis of the convergence below.

# B Backward shifts of successor features

Here, as mentioned in subsection 5.5, we want to prove backward shifting of successor features which are Gaussian, but let us first make some general remarks. Recall that we are assuming an idealization of a linear track as the real line $\mathbb{R}$, where an agent is moving on. We are considering a feature $\phi : \mathbb{R} \to \mathbb{R}$ and want to study the shift of it's center of mass, defined as the normalized mean $COM = \frac{\int x\phi(x)dx}{\int \phi(x)dx}$

Since the feature in our case is obtained as inputs from other neurons, it makes sense to assume that $\phi$ is non-negative. Without loss of generality, we also assume that the feature is normalized, that is $\phi$ is a probability density, and the center of mass simply becomes $\int x\phi(x)dx = \mathbb{E}_\phi[x]$. Furthermore, when we normalize the successor operator (which is simply the Laplace transform) by multiplying by $\gamma$, that is by letting

$$SF(\phi)(x) = \int_0^\infty \int \gamma e^{-\gamma t} \phi(y) p_t(y|x) dy dt, \tag{21}$$

then we have that $SF(\phi)(x) = \mathbb{E}_{q(y|x)}[\phi(y)]$, with $q(y|x) = \int_0^\infty \gamma e^{-\gamma t} p_t(y|x)dt$ the density obtained from transforming $p_t(y|x)$ (one can think of this as the transition density from $x$ to $y$ when time $t$ is sampled from an exponential distribution). In general, it might be difficult to study the centre of mass, because for example $SF$ is not necessarily normalized anymore.

However, the case we consider is more benign, so let us turn to the special case now after these general remarks. Assume that we have Gaussian features, that is

$$\phi(x) = \mathcal{N}(x; \mu, \sigma) := \frac{1}{\sqrt{2\pi\sigma}} \exp\left(-\frac{(x-\mu)^2}{2\sigma}\right). \tag{22}$$

Hence, the centre of mass is simply the mean $\mu$. Let the dynamics be a Brownian motion with a constant drift $v$, that is

$$p_t(y|x) = \mathcal{N}\left(y; x + vt, \rho\sqrt{t}\right). \tag{23}$$

In particular, as a function of $x$ we can write this as

$$\mathcal{N}\left(x; y - vt, \rho\sqrt{t}\right). \tag{24}$$

Then integrating the transition density and $\phi$, just yields the marginal distribution, which by the well known properties of Gaussians is again a Gaussian

$$\int p_t(y|x)\phi(y) = \mathcal{N}\left(x; \mu - vt, \rho\sqrt{t} + \sigma\right). \tag{25}$$

Thus, the successor feature is then given by

$$SF(\phi)(x) = \int_0^\infty \int \gamma e^{-\gamma t}\phi(y)p_t(y|x)dydt \tag{26}$$

$$= \int_0^\infty \gamma e^{-\gamma t}\mathcal{N}\left(x; \mu - vt, \rho\sqrt{t} + \sigma\right) dt. \tag{27}$$

In particular, the centre of mass is

$$\mathbb{E}[SF(\phi)(x)] = \int_0^\infty \gamma e^{-\gamma t} \int \mathcal{N}\left(x; \mu - vt, \rho\sqrt{t} + \sigma\right) dxdt \tag{28}$$

$$= \int_0^\infty \gamma e^{-\gamma t}(\mu - vt) \tag{29}$$

$$= \mu - \frac{v}{\gamma}. \tag{30}$$

Thus, we observe again, just as in the example considered in the main text, a shift which depends on the predictive time scale factor $\gamma$ and the velocity $v$ - with a bigger $\gamma$ or a smaller $v$ leading to less shift. In particular, if the velocity is zero, then no shift should be observed.

# C Eigenvectors of the Successor Representation

The eigenvectors of the successor representation matrix $(\mathrm{Id} - \gamma P)^{-1}$ are of special importance in the theory both in computational neuroscience as well as in reinforcement learning. In the latter, these eigenvectors are used to construct a natural basis for functions on the state space, which yield a representation of the large-scale geometry of the state space and can be used for example for option discovery [4–6]. In neuroscience in turn, these eigenvectors have been used as a model of grid cells, since in spatial settings there are remarkable similarities of some of the eigenvectors to grid cells observed in the entorhinal cortex [7, 8]. The key insight to why these eigenvectors are particularly useful to represent the state space is that they are essentially the eigenvectors of a Laplace operator [7]. On an undirected graph with adjacency matrix $A$, the random-walk Laplacian is defined as

$$\mathrm{Id} - D^{-1}A \tag{31}$$

where $D$ is the diagonal matrix with entries $D_{ii} = \frac{1}{\sum_j A_{ij}}$. There are other ways to define a Laplacian [9], but on an undirected graph they are all related [10]. In Equation 31, the matrix $P := D^{-1}A$ corresponds to the transition probability matrix of arandom walk where the neighbors of a vertex are visited with the same probability. It is then not hard to see that the random walk Laplacian and the successor representation under $P$ share the same eigenvectors (although for different eigenvalues). These eigenvectors and associated eigenvalues have many desirable properties: they are invariant under certain symmetries (automorphisms) of the graph [6], they can be used to cluster the graph [11] and they represent slowly varying/smooth features [12]. Many of the technical results on graph Laplacians hinge on self-adjointness (i.e., symmetry), which is why they do not directly translate to the setting of directed graphs. In this situation, it is thus common to include some form of symmetrization - in fact, from the beginning in RL it was proposed to use Laplacian eigenfunctions under a symmetrization [6]. There are at least two straightforward ways how to do this: either, one can treat each directed edge as an undirected edge - this corresponds to constructing a new adjacency matrix $\frac{1}{2}(A + A^T)$, and was for example used in [6] to obtain basis functions for RL. The other possibility is to use the information from the stationary distribution, and symmetrize as $\frac{1}{2}(P + \Pi^{-1}P^T\Pi)$ - this leads to the so called 'Chung-Laplacian' [13]; also this Laplacian construction has been successfully applied in RL [14, 15]. We have seen that our symmetrized learning rule in the stationary setting will learn the successor representation under $P + \Pi^{-1}P^T\Pi$, and therefore eigenvalues constructed from it would correspond to those of the Chung-Laplacian. However, in practice stationarity might not be a valid assumption - this is for example the case in our simulations, where episodes are terminated on reward. In this case, the symmetric representation that is learned is actually more closely related to the former case.

# D Convergence proofs

In the following, we will give convergence proofs for both the neural network model and the TD-learning setting. In both situations, we will use the same strategy as in [16]. Therein, they use a classical result from stochastic approximation theory [17]. We state now a formulation of this result close to the one used in [16], but note that this omits some detail.

**Theorem 1.** *Let $Z_n \in \mathbb{R}^n$ be a sequence of random variables, which fulfill*

$$Z_{n+1} = Z_n + \varepsilon_n k(Z_n), \tag{32}$$

*where $k$ is a stochastic function of $Z$ mapping to $\mathbb{R}^n$, and $\varepsilon_n \in (0,1)$ is a sequence such that $\sum_n \varepsilon_n = \infty, \sum_n \varepsilon_n^2 < \infty$. Define $\overline{k} : z \mapsto \mathbb{E}[k(z)]$, assume that $Z_n$ is bounded almost surely and that $\mathrm{Var}[k(Z_n) - \overline{k}(Z_n)]$ is bounded. Let $A^0$ be the set of asymptotically stable equillibria of the differential equation*

$$\frac{d}{dt}z(t) = \overline{k}(z). \tag{33}$$

*Then if $A^0$ is nonempty, $Z_n \to A^0$.*

As in [16], we will not verify the boundedness conditions, because these could be enforced by projecting the sequence back into a bounded region if necessary - i.e., one could modify the update equations to include a projection step that enforces staying in a certain bounded region. Then, all quantities above are bounded and hence this new sequence converges to $A^0$ if $A^0$ is in that region.

## D.1 Convergence of Model

In the following, to avoid using too many superscripts, we change notation compared to the main text, and set $W = W^1$ the recurrent weight,$V = W^2$ the feedforward weight, $p = p^1, q = p^2$ the population activities, $\phi = \phi^1, \psi = \phi^2$ the inputs. Furthermore, we use the notation

$$R(W) = (Id - \gamma W)^{-1}$$

and recall our definition

$$P_{\alpha,\beta} = \frac{\alpha}{\alpha+\beta} P^T + \frac{\beta}{\alpha+\beta} \Pi P \Pi^{-1}.$$

Furthermore, recall that we assume our neural activities $p, q$, given inputs and synaptic weights are

$$p_t = R(W)\phi_t \tag{34}$$
$$q_t = \gamma V p_t + \psi_t \tag{35}$$

Consider the update rule for the system

$$W_{t+1} = W_t + \varepsilon_t \Delta_{W,t} \tag{36}$$
$$V_{t+1} = V_t + \varepsilon_t \Delta_{V,t} \tag{37}$$
$$\Delta_{W,t} = (\alpha_W (p_{t+1} - W_t p_t) p_t^T + \beta_W (p_t - W_t p_{t+1}) p_{t+1}^T). \tag{38}$$
$$\Delta_{V,t} = (\alpha_V (q_{t+1} - V_t p_t) p_t^T + \beta_V (q_t - V_t p_{t+1}) p_{t+1}^T), \tag{39}$$

As stated in the theorem above, we can show convergence to $W^*, V^*$ if we can show that the differential equation

$$\frac{d}{dt}\begin{pmatrix} W \\ V \end{pmatrix} = \mathbb{E}\left[\begin{pmatrix} \Delta(W) \\ \Delta(V) \end{pmatrix}\right] \tag{40}$$

has an asymptotically stable fixed point at $W^*, V^*$ - to do so, we will study eigenvalues of the Jacobian at putative fixed points. Recall that for an autonomous dynamical system $dx/dt = F(x)$ and some fixed point $x_0$, if the linearized version $du/dt = Df(x_0)(u)$ of the system is asymptotically stable at $x_0$, so is the original system. In turn, if the Jacobian $DF(x_0)$ of the system at that fixed point has eigenvalues with all negative real parts, the linearized system is asymptotically stable. This lays down our strategy to prove stability.

First, we compute the right hand side of the differential equation Equation 40. We have approximately (assuming for the expectation that $W_{t+1} \approx W_t$, $V_{t+1} \approx V_t$):

$$\Delta(W) = \alpha_W \left(R(W)\phi_{t+1} - WR(W)\phi_t\right) \phi_t^T R(W)^T + \tag{41}$$
$$\beta_W \left(R(W)\phi_t - WR(W)\phi_{t+1}\right) \phi_{t+1}^T R(W)^T \tag{42}$$

and (recall $q = \gamma_V V p + \psi$)

$$\Delta(V) = \alpha_V \left(V\left(\gamma_V R(W)\phi_{t+1} - R(W)\phi_t\right) + \psi_{t+1}\right) \phi_t^T R(W) + \tag{43}$$
$$\beta_V \left(\gamma_V V\left(R(W)\phi_t - R(W)\phi_{t+1}\right) + \psi_t\right) \phi_{t+1}^T R(W)^T \tag{44}$$

We now assume that the inputs $\psi_t, \phi_t$ are in stationary distribution (we state below a condition under which this holds) and then take the expectation to obtain

$$\mathbb{E}[\Delta(W)] = \left(R(W)\left(\alpha\mathbb{E}[\phi_1\phi_0^T]] + \beta\mathbb{E}[\phi_0\phi_1^T]] - (\alpha+\beta)W\mathbb{E}[\phi_0\phi_0^T]\right) R(W)^T \tag{45}$$

$$\mathbb{E}[\Delta(V)] = VR(W)\left(\gamma_V(\alpha_V\mathbb{E}[\phi_1\phi_0^T] + \beta_V\mathbb{E}[\phi_0\phi_1^T]) - (\alpha_V + \beta_V)\mathbb{E}[\phi_0\phi_0^T]\right) R(W)^T \tag{46}$$
$$+ (\alpha_V\mathbb{E}[\psi_1\phi_0^T] + \beta_V\mathbb{E}[\psi_0\phi_1^T])R(W)^T \tag{47}$$

where we have used that $W$ and $R(W)$ commute. We now will find the putative fixed points oft these equations by finding their zeros. For the first equation, setting the innermost term zero, we see that this equation has an equilibrium at $W^*$ if

$$W^*\mathbb{E}[\phi_0\phi_0^T] = \frac{\alpha_W}{\alpha_W + \beta_W}\mathbb{E}[\phi_1\phi_0^T] + \frac{\beta_W}{\alpha_W + \beta_W}\mathbb{E}[\phi_0\phi_1^T] \tag{48}$$

We see that this equation essentially prescribes $W$ to implement a weighted average of the linear regression of $\phi_0$ against $\phi_1$ and vice versa. Indeed, if $\mathbb{E}[\phi_0\phi_0^T]$ is invertible one sees that it becomes

$$W^* = \frac{\alpha_W}{\alpha_W + \beta_W}\mathbb{E}[\phi_1\phi_0^T]\mathbb{E}[\phi_0\phi_0^T]^{-1} + \frac{\beta_W}{\alpha_W + \beta_W}\mathbb{E}[\phi_0\phi_1^T][\phi_1\phi_1^T]^{-1}, \tag{49}$$

and $[\phi_1\phi_0^T]\mathbb{E}[\phi_0\phi_0^T]^{-1}$ is for example the regression weight for linearly predicting $\phi_1$ from $\phi_0$.

Setting now the second equation to zero yields the relation which has to hold at a putative fixed point $V*$:

$$V^*R(W)\left(\mathbb{E}[\phi_0\phi_0^T] - \gamma_V\left(\frac{\alpha_V}{\alpha_V + \beta_V}\mathbb{E}[\phi_1\phi_0^T] + \frac{\beta_V}{\alpha_V + \beta_V}\mathbb{E}[\phi_0\phi_1^T]\right)\right) = \frac{\alpha_V}{\alpha_V + \beta_V}\mathbb{E}[\psi_1\phi_0^T] + \frac{\beta_V}{\alpha_V + \beta_V}\mathbb{E}[\psi_0\phi_1^T]. \tag{50}$$

We now want perform a stability analysis of the equations at an equillibrium $(W^*, V^*)$. Since the dynamics of $W$ do not depend on $V$, we can look at the

respective equations separately and if both are stable, the whole system is stable. The easier part is the equation for $V$: It is affine linear in $V$, that is we only have to study the eigenvalues of the matrix

$$R(W^*)\left(\gamma_V(\alpha_V\mathbb{E}[\phi_1\phi_0^T]+\beta_V\mathbb{E}[\phi_0\phi_1^T])-(\alpha_V+\beta_V)\mathbb{E}[\phi_0\phi_0^T]\right)R(W^*)^T \tag{51}$$

In fact, using lemma 3, we only have to study the eigenvalues of the inner term $\left(\gamma_V(\alpha_V\mathbb{E}[\phi_1\phi_0^T]+\beta_V\mathbb{E}[\phi_0\phi_1^T])-(\alpha_V+\beta_V)\mathbb{E}[\phi_0\phi_0^T]\right)$. According to lemma 6, these have all negative real parts, hence we have stability for this equation at any equilibrium.

The stability of the differential equation for $W$ is slightly more delicate. Let $F$ denote the right hand side of the differential equation for $W$. We want to find its Jacobian. First, we want to calculate the derivative of $F$ (in the Frechet sense) at $W^*$, as a linear map applied to a matrix $U$. We have that

$$D_R(W)(U)=-R(W)\gamma UR(W) \tag{52}$$

and thus by the chain rule

$$\begin{aligned}
DF_{W^*}(U) = &-R(W^*)\gamma UR(W^*)\left(\alpha\mathbb{E}[\phi_1\phi_0^T]+\beta\mathbb{E}[\phi_0\phi_1^T]-(\alpha+\beta)W^*\mathbb{E}[\phi_0\phi_0^T]\right)R(W^*) && (53)\\
&-R(W^*)\left(\alpha\mathbb{E}[\phi_1\phi_0^T]+\beta\mathbb{E}[\phi_0\phi_1^T]-(\alpha+\beta)W^*\mathbb{E}[\phi_0\phi_0^T]\right)R(W^*)^T\gamma U^TR(W^*)^T && (54)\\
&-R(W^*)(\alpha+\beta)U\mathbb{E}[\phi_0\phi_0^T]R(W^*)^T && (55)\\
&=-R(W^*)(\alpha+\beta)U\mathbb{E}[\phi_0\phi_0^T]R(W^*)^T, && (56)
\end{aligned}$$

where the first two terms vanished at $W^*$ because by assumption $\left(\alpha\mathbb{E}[\phi_1\phi_0^T]]+\beta\mathbb{E}[\phi_0\phi_1^T]]-(\alpha+\beta)W^*\mathbb{E}[\phi_0\phi_0^T]\right)=0$.

Now, we want to obtain the Jacobian from the expression above, that is we want to find a matrix that represents this derivative. To achieve this, we may vectorize the matrix $U$ using the vectorization operator vec, which transforms a matrix into a vector by stacking the columns on top of each other. Using the well known identity

$$\operatorname{vec}(ABC)=(C^T\otimes A)\operatorname{vec}(B), \tag{57}$$

where $\otimes$ denotes the Kronecker product of matrices, we thus have

$$-\operatorname{vec}R(W^*)(\alpha+\beta)U\mathbb{E}[\phi_0\phi_0^T]R(W^*)^T=-(\alpha+\beta)(\mathbb{E}[\phi_0\phi_0^T]R(W^*)\otimes R(W^*))\operatorname{vec}(U). \tag{58}$$

This shows that the Jacobian is given by the matrix

$$M=-(\alpha+\beta)R(W^*)\mathbb{E}[\phi_0\phi_0^T]\otimes R(W^*). \tag{59}$$

Now, for the Kronecker-product of two matrices, the eigenvalues are given as the products of the eigenvalues of the individual matrices. That is, if $\lambda_i$ are

the eigenvalues of $R(W^*)\mathbb{E}[\phi_0\phi_0^T]$ and $\mu_j$ are the eigenvalues of $R(W^*)$, the eigenvalues of $M$ are given by

$$-(\alpha+\beta)\lambda_i\mu_j. \tag{60}$$

Now there seems to be in general *no* guarantee that these all have negative real parts and thus the matrix $M$ be stable for all choices of $\alpha, \beta, \phi$.

However, we can obtain a results in the symmetric case, under some assumptions (which will hold in the case we are interested in):

**Proposition 1.** *For $\alpha = \beta > 0$, $\mathbb{E}[\phi_0\phi_0^T]$ invertible, and the real part of any eigenvalue $\mu$ of $W^*$ fulfilling $\mathrm{Re}(\mu) \leq 1$ the matrix $M$ is stable, that is all eigenvalues have negative real parts.*

*Proof.* First, we want to consider the eigenvalues of $R(W^*)\mathbb{E}[\phi_0\phi_0^T]$. We note that by the defining relation of $W^*$ as a fixed point we obtain that

$$R(W^*)\mathbb{E}[\phi_0\phi_0^T] = R(W^*)\left(\mathbb{E}[\phi_0\phi_0^T] - \gamma\left(\frac{\alpha_W}{\alpha_W+\beta_W}\mathbb{E}[\phi_1\phi_0^T] + \frac{\beta_W}{\alpha_W+\beta_W}\mathbb{E}[\phi_0\phi_1^T]\right)\right)^T R(W^*)^T. \tag{61}$$

According to lemma 6 and 3, the eigenvalues of the right hand side have positive real parts, and for $\alpha = \beta$, the matrix is symmetric hence the eigenvalues are positive reals. On the other hand, the eigenvalues of the matrix $R(W)^* = (\mathrm{Id} - \gamma W^*)^{-1}$ have positive real parts by assumption and lemma 4. Thus, also the products of the eigenvalues of the two matrices have positive real parts, which proves the claim. □

We have thus confirmed that in the symmetric case, it is possible to have stability (and thus convergence) at some $(W^*, V^*)$. Below, we will now assume a specific structure of the inputs which will link to successor features and which will make it possible to state the existence of a solution. We will now assume

$$\phi_t = \tilde{\phi}(S_{t+\tau_1}) = \Phi e_{S_{t+\tau_1}} \tag{62}$$

$$\psi_t = \tilde{\psi}(S_{t+\tau_2}) = \Psi e_{S_{t+\tau_2}} \tag{63}$$

where $S_t$ is the state process, which we assume in stationary distribution, $\tilde{\phi}, \tilde{\psi}$ are injective functions, the matrices $\Psi, \Phi$ have the respective values of these functions as their columns, The $\tau_i$ indicate that the two inputs could have a temporal offset, we will however set these to zero in the sequel. We note at these points that these choices are made to relate to the definition successor features, and other choices of input could equally yield a similar result.

With the above definitions, we have

$$\mathbb{E}[\phi_0\phi_0^T] = \Phi\Pi\Phi^T \tag{64}$$

$$\mathbb{E}[\phi_1\phi_0^T] = \Phi P^T\Pi\Phi^T \tag{65}$$

$$\mathbb{E}[\phi_0\phi_1^T] = \Phi\Pi P\Phi^T \tag{66}$$

$$\mathbb{E}[\psi_1\phi_0^T] = \Psi P^T\Pi\Phi^T \tag{67}$$

$$\mathbb{E}[\psi_0\phi_1^T] = \Psi\Pi P\Phi^T \tag{68}$$

where $P$ is the transition matrix of $S_t$ and $\Pi$ is again the diagonal matrix of the stationary distribution. Plugging these expressions into the relation we derived for $W^*$ yields

$$W^*\Phi\Pi\Phi^T = \frac{\alpha_W}{\alpha_W+\beta_W}\Phi P^T\Pi\Phi^T + \frac{\beta_W}{\alpha_W+\beta_W}\Phi\Pi P\Phi^T \tag{69}$$
$$= \Phi P^T_{\alpha_W,\beta_W}\Pi\Phi^T \tag{70}$$
$$\tag{71}$$

where it is now easy to see that all possible solutions for $W^*$ are of the form

$$W^* = \Phi P^T_{\alpha_W,\beta_W}\Phi^- + C \tag{72}$$

where $\Phi^-$ denotes the pseudoinverse, and $C$ is a matrix with $C\Psi = 0$.

Similarly, for $V^*$ we obtain the relation

$$V^*R(W)\left(\Phi\Pi\Phi^T - \gamma_V\left(\frac{\alpha_V}{\alpha_V+\beta_V}\Phi P^T\Pi\Phi^T + \frac{\beta_V}{\alpha_V+\beta_V}\Phi\Pi P\Phi^T\right)\right) \tag{73}$$
$$= \frac{\alpha_V}{\alpha_V+\beta_V}\Psi P^T\Pi\Phi^T + \frac{\beta_V}{\alpha_V+\beta_V}\Psi\Pi P\Phi^T. \tag{74}$$

which may be written as

$$V^*R(W)\left(\Phi\left(\mathrm{Id} - \gamma_V\Phi P^T_{\alpha_V,\beta_V}\right)\Pi\Phi^T\right) = \Psi P^T_{\alpha_V,\beta_V}\Pi\Phi^T \tag{75}$$

It is then again clear that all possible solutions for $V^*$ are of the form

$$V^* = \left(\Psi P^T_{\alpha_V,\beta_V}(\mathrm{Id} - \gamma_V P^T_{\alpha_W,\beta_W})^{-1}\Phi^- + C\right)R(W^*)^{-1} \tag{76}$$

where $C$ is again an arbitrary matrix having $\Phi$ in its kernel.

We note that with this particular form of $W^*$, we can indeed achieve stable solutions: $\Phi P_{\alpha_W,\beta_W}\Phi^-$ has eigenvalues either being zero or corresponding to eigenvalues of $P_{\alpha_W,\beta_W}$, which is a stochastic matrix and hence has eigenvalues in the unit circle, such that as long as the matrix $C$ has eigenvalues with real part lesser or equal than 1, the requirements of our proposition above are met and we have a stable equilibrium. In the particular special case where $\Phi$ is invertible, we get unique stable solutions.

### D.1.1 Limits of activities

With these particular solutions, we can understand the form the activities of the populations will take. We will plug in the limits of the weight matrices into the equations

$$p_t = (\mathrm{Id} - \gamma_W W^*)^{-1}\phi_t \tag{77}$$
$$q_t = \gamma_V V^* p_t + \psi_t \tag{78}$$

First, we recall that we assume inputs of the form $\phi_t = \phi(S_t)$. Second, we note that the action of the matrices on the space spanned by $\Phi$ is the same for

all solutions $W^*$, regardless of the additional matrix $C$: As $C\Phi = 0$, whenever our input is in the space spanned by the features, we have $W^*\phi_t = \Phi P_{\alpha,\beta}^T \Phi^-$. Now, since $\Phi^-$ acts as an inverse on this space, we have that

$$\Phi^- \phi(S_t) = e_{S_t}. \tag{79}$$

Hence, in particular

$$\Phi P_{\alpha,\beta^T} \Phi^- \phi_t = \Phi P_{\alpha,\beta}^T e_{S_t} = \sum_{s'} \phi(s') P_{\alpha,\beta}(s'|S_t). \tag{80}$$

Thus, we have shown that

$$\Phi P_{\alpha,\beta^T} \Phi^- \phi_t = \mathbb{E}_{P_{\alpha,\beta}^T}[\phi_{t+1}|\phi_t] = \mathbb{E}_{P_{\alpha,\beta}^T}[\phi_{t+1}|S_t]. \tag{81}$$

The same arguments holds for any powers of the matrix $W^*$: Indeed, taking

$$(\Phi P_{\alpha_W,\beta_W} \Phi^- + C)^k \tag{82}$$

will result in a sum of mixed terms of powers of the two matrices in the sum. However, whenever $C$ appears to the left of $\Phi$, the matrix will be zero, and whenever $C$ is the rightmost term, the matrix again has the span of the columns of $\Phi$ in its kernel. Thus, one may consider only the terms

$$(\Phi P_{\alpha_W,\beta_W} \Phi^-)^k + C^k \tag{83}$$

and then perform exactly the same calculation as above. This then yields

$$p_t = (\mathrm{Id} - \gamma_W W^*)^{-1} \phi_t \tag{84}$$

$$= \left(\mathrm{Id} - \gamma_W \Phi P_{\alpha_W,\beta_W}^T \Phi^-\right)^{-1} \phi_t \tag{85}$$

$$= \sum_k \gamma_W^k \mathbb{E}_{P_{\alpha,\beta}} \left[\phi(S_{t+k})|S_t\right] \tag{86}$$

$$= SR_\phi^{P_{\alpha_W,\beta_W}}(S_t) \tag{87}$$

which shows that indeed $p_t$ encodes the successor representation of the function $\phi$.

Now for $q_t$,first recall that for $V^*$ we obtained the limit

$$\left(\Psi P_{\alpha_V,\beta_V}^T (\mathrm{Id} - \gamma_V P_{\alpha_W,\beta_W}^T)^{-1} \Phi^- + C\right) R(W^*)^{-1} \tag{88}$$

where $C$ is a matrix with the span of $\Phi$ in its kernel and $R(W^*) = (\mathrm{Id} - \gamma_W W^*)^{-1}$. Now plugging this in into the equation for $q_t$ yields

$$q_t = \gamma_V V^* p_t + \psi_t \tag{89}$$

$$= \gamma_V \left(\Psi P_{\alpha_V,\beta_V}^T (\mathrm{Id} - \gamma_V P_{\alpha_W,\beta_W}^T)^{-1} \Phi^- + C\right) R(W^*)^{-1} p_t + \psi_t \tag{90}$$

$$= \gamma_V \left(\Psi P_{\alpha_V,\beta_V}^T (\mathrm{Id} - \gamma_V P_{\alpha_W,\beta_W}^T)^{-1} \Phi^- + C\right) R(W^*)^{-1} R(W^*) \phi_t + \psi_t \tag{91}$$

$$= \gamma_V \left(\Psi P_{\alpha_V,\beta_V}^T (\mathrm{Id} - \gamma_V P_{\alpha_W,\beta_W}^T)^{-1} \Phi^- + C\right) \phi_t + \psi_t \tag{92}$$

$$= \gamma_V \left(\Psi P_{\alpha_V,\beta_V}^T (\mathrm{Id} - \gamma_V P_{\alpha_W,\beta_W}^T)^{-1} \Phi^-\right) \phi_t + \psi_t. \tag{93}$$

Then, note that

$$\operatorname{Id} + \gamma_V P^T_{\alpha_V,\beta_V} \left(\operatorname{Id} - \gamma_V P^T_{\alpha_V,\beta_V}\right)^{-1} = \left(\operatorname{Id} - \gamma_V \; P^T_{\alpha_V,\beta_V}\right)^{-1} \tag{94}$$

as can be seen immediately by multiplying both sides by $\left(\operatorname{Id} - \gamma_V P^T_{\alpha_V,\beta_V}\right)$. Further note that $\Phi^- \phi_t = \Psi^- \psi_t = e_{S_t}$ by definition. Using this, we have

$$\begin{aligned} q_t &= \gamma_V \Psi P^T_{\alpha_V,\beta_V} \left(\operatorname{Id} - \gamma_V P^T_{\alpha_V,\beta_V}\right)^{-1} \Phi^- \phi_t + \psi_t && (95)\\ &= \Psi \left(\operatorname{Id} + \gamma_V P^T_{\alpha_V,\beta_V} \left(\operatorname{Id} - \gamma_V P^T_{\alpha_V,\beta_V}\right)^{-1}\right) \Psi^- \psi_t && (96)\\ &= \Psi \left(\operatorname{Id} - \gamma_V \; P^T_{\alpha_V,\beta_V}\right)^{-1} \Psi^- \psi_t && (97)\\ &= \sum_k \gamma_V^k \mathbb{E}_{P_{\alpha_V,\beta_V}} \left[\psi(S_{t+k}) | S_t\right] && (98)\\ &= SR_\psi^{P_{\alpha_V,\beta_V}}(S_t) && (99) \end{aligned}$$

which shows that also $q$ encodes a succesor representation.

# E Convergence of TD learning

Here we show in a very similar manner that classical TD-learning with the inclusion of parameters $\alpha, \beta$ learns the successor representation under the modified probabilities. In fact, one can see classical TD-learning as a one layer feedforward network implementing the learning rule/

Consider the following modified TD-learning rule for the parameter $\theta_t$ parametrizing $\tilde{V}$

$$\begin{aligned} \theta_{t+1} &= \theta_t + \varepsilon_t \Delta_t && (100)\\ \Delta_t &= \alpha \left(\phi(S_t) + \gamma \tilde{V}(\theta_t, S_{t+1}) - \tilde{V}(\theta_t, S_t)\right)^T \nabla_\theta \tilde{V}(\theta_t, S_t) && (101)\\ &\quad + \beta \left(\phi(S_{t+1}) + \gamma \tilde{V}(\theta_t, S_t) - \tilde{V}(\theta_t, S_{t+1})\right)^T \nabla_\theta \tilde{V}(\theta_t, S_{t+1}). && (102) \end{aligned}$$

We can see this as a semi-gradient descent with respect to the loss

$$L(s, s', \theta, \theta') = \alpha ||\phi(s) + \gamma \tilde{V}(\theta', s') - \tilde{V}(\theta, s)||^2 + \beta ||\phi(s') + \gamma \tilde{V}(\theta', s) - \tilde{V}(\theta, s')||^2. \tag{103}$$

Now assume the linear and tabular case, that is

$$\begin{aligned} \tilde{V}(W, s) &= W e_s, \phi(S) = \Phi e_s && (104)\\ W_{t+1} &= W_t + \varepsilon_t \Delta_t && (105)\\ \Delta_t &= \alpha \left(\Phi e_{S_t} + \gamma W_t e_{S_{t+1}} - W_t e_{S_t}\right) e_{S_t}^T && (106)\\ &\quad + \beta \left(\Phi e_{S_{t+1}} + \gamma W_t e_{S_t} - W_t e_{S_{t+1}}\right) e_{S_{t+1}}^T && (107) \end{aligned}$$

Note that we can also write the updates as

$$W_{t+1}(ij) = (1 - \epsilon_t(ij))W_t(ij) \tag{108}$$
$$+ \varepsilon_t(ij) \left( \alpha \left( \Phi e_{S_t} e_{S_t}^T + \gamma W_t e_{S_{t+1}} e_{S_t}^T \right) + \beta \left( \Phi e_{S_{t+1}} e_{S_{t+1}}^T + \gamma W_t e_{S_t} e_{S_{t+1}}^T \right) \right) (ij) \tag{109}$$

where $\epsilon_t(ij) = \varepsilon_t(\alpha \mathbb{I}[S_t = j] + \beta \mathbb{I}[S_{t+1} = j])$.

We have

$$\mathbb{E}[\Delta(W)] = \mathbb{E}[\alpha \left( \Phi e_{S_t} + \gamma W e_{S_{t+1}} - W e_{S_t} \right) e_{S_t}^T \tag{110}$$
$$+ \beta \left( \Phi e_{S_{t+1}} + \gamma W e_{S_t} - W e_{S_{t+1}} \right) e_{S_{t+1}}^T .] \tag{111}$$
$$= \alpha(\Phi\Pi + \gamma W P^T \Pi - W\Pi) \tag{112}$$
$$+ \beta(\Phi\Pi + \gamma W \Pi P - W\Pi) \tag{113}$$
$$= (\alpha + \beta) \left( \Phi - W \left( \mathrm{Id} - \gamma \left( (\frac{\alpha}{\alpha+\beta} P^T + \frac{\beta}{\alpha+\beta} \Pi P \Pi^{-1} \right) \right) \right) \Pi. \tag{114}$$

Setting to zero, we obtain

$$W^* = \Phi \left( \mathrm{Id} - \gamma \left( (\frac{\alpha}{\alpha+\beta} P^T + \frac{\beta}{\alpha+\beta} \Pi P \Pi^{-1} \right) \right)^{-1}, \tag{115}$$

which is precisely the successor representation under $P_{\alpha,\beta}$. The stability of the differential equation, which is linear, is determined by the eigenvalues of the matrix

$$M = \left( \mathrm{Id} - \gamma \left( \frac{\alpha}{\alpha+\beta} P^T + \frac{\beta}{\alpha+\beta} \Pi P \Pi^{-1} \right) \right) \Pi \tag{116}$$

Using again lemma 6 below this has all positive eigenvalues, which proves the stability and hence the convergence.

# F Auxiliary results

Here we state some lemmas we used for the convergence proofs above, these should all be standard results, but we prove them here for convenience.

**Lemma 2.** *Let $M \in \mathbb{R}^{n \times n}$ (not necessarily symmetric). Then if $M$ is positive definite in the sense that for any $0 \neq x \in \mathbb{R}^n$, $x^T M x > 0$, all eigenvalues of $M$ have positive real part.*

*Proof.* Let $\lambda \in \mathbb{C}$ be an eigenvalue of $M$ with eigenvector $z \in \mathbb{C}^n$. Then on the one hand we have

$$\langle z, Mz \rangle_{\mathbb{C}^n} = \langle z, \lambda z \rangle_{\mathbb{C}^n} = \lambda \langle z, z \rangle_{\mathbb{C}^n}, \tag{117}$$

and on the other hand

$$\operatorname{Re}\left(\langle z, Mz\rangle_{\mathbb{C}^n}\right) = \sum_{i,j} \operatorname{Re}(z_i) M_{ij} \operatorname{Re}(z_j) + \sum_{i,j} \operatorname{Im}(z_i) M_{ij} \operatorname{Im}(z_j) \tag{118}$$

Now since $\langle z, z\rangle_{\mathbb{C}^n} > 0$, if $M$ is positive definite, the right hand side of the last equation is positive and this implies that $Re(\lambda) > 0$. □

**Lemma 3.** *Let $A, B$ be in $\mathbb{R}^{n\times n}$ and $B$ be invertible. Then $A$ is positive definite in the sense of the previous lemma if and only if so is $BAB^T$.*

*Proof.* Since $B$ is invertible,

$$x^T Ax > 0 \forall x \iff (By)^T A(By) = y^T B^T ABy > 0 \forall y. \tag{119}$$

□

**Lemma 4.** *Let $Q$ be a matrix $Re(\lambda) < 1$ for all its eigenvalues $\lambda \in \mathbb{C}$. Then $Id - Q$ has eigenvalues with all positive real parts, is invertible, and the inverse also has eigenvalues with all positive real parts.*

*Proof.* The eigenvalues of $Id - Q$ are of the form $1 - \lambda$. Hence we have $\operatorname{Re}(1-\lambda) > 0$. This then implies that $Id - Q$ is invertible. The last part follows since inversion maps the positive half-plane to the positive half-plane. □

**Lemma 5.** *Let $A$ be a square matrix and $A^*$ be it's adjoint with respect to some inner product $\langle\cdot,\cdot\rangle$. Then we have, denoting by $\sigma$ the spectrum,*

$$\max_{\lambda\in\sigma(A)} Re(\lambda) \le \max_{\mu\in\sigma(\frac{1}{2}(A+A^*))} \mu. \tag{120}$$

*Proof.* We note that for an eigenpair $(v, \lambda)$ of $A$

$$\langle v, (A + A^*)v\rangle = \langle v, Av\rangle + \langle Av, v\rangle = (\lambda + \overline{\lambda})\langle v, v\rangle = 2Re(\lambda)\langle v, v\rangle \tag{121}$$

Hence, by the Courant-principle

$$Re(\lambda) = \frac{\langle v, \frac{1}{2}(A + A^*)v\rangle}{\langle v, v\rangle} \le \max_x \frac{\langle x, \frac{1}{2}(A + A^*)x\rangle}{\langle x, x\rangle} = \max_{\mu\in\sigma(\frac{1}{2}(A+A^*)} \mu. \tag{122}$$

□

**Corollary 1.** *Let $t \in \mathbb{R}$, $P$ a stochastic matrix with stationary distribution $\pi$, and $\Pi = \operatorname{diag}(\pi)$. The matrix*

$$Q = tP + (1 - t)\Pi^{-1}P^T\Pi \tag{123}$$

*has $Re(\lambda) \le 1$ for all its eigenvalues $\lambda \in \mathbb{C}$.*

*Proof.* Consider the inner product $\langle x, x\rangle_\Pi = x^T \Pi x$. With respect to this inner product, the adjoint of $Q$ is

$$Q^* = (1-t)P + t\Pi^{-1}P^T\Pi. \tag{124}$$

Hence,

$$\frac{1}{2}(Q + Q^*) = \frac{1}{2}(P + \Pi^{-1}P^T\Pi) \tag{125}$$

which is a stochastic matrix and therefore has largest eigenvalue 1. □

**Lemma 6.** *Let $\phi_0, \phi_1$ be random vectors distributed equally. Let furthermore $t \in \mathbb{R}$ and $\gamma \in [0, 1)$. Then the matrix*

$$M = \mathbb{E}[\phi_0\phi_0^T] - \gamma\left(t\mathbb{E}[\phi_1\phi_0^T] + (1-t)\mathbb{E}[\phi_0\phi_1^T]\right) \tag{126}$$

*has eigenvalues with all nonnegative real parts. If furthermore $\mathbb{E}[\phi_0\phi_0^T]$ has full rank, then the real parts are all positive.*

*Proof.* Let $x$ be any nonzero vector. Note that we have

$$x^T\mathbb{E}[(\phi_1 - \phi_0)(\phi_1 - \phi_0)^T]x = x^T \operatorname{Cov}[\phi_1 - \phi_0]x \geq 0, \tag{127}$$

hence

$$2x^T\mathbb{E}[\phi_0\phi_0^T]x = x^T\mathbb{E}[\phi_0\phi_0^T] + x^T\mathbb{E}[\phi_1\phi_1^T]x^T \geq x^T\mathbb{E}[\phi_1\phi_0^T]x + x^T\mathbb{E}[\phi_0\phi_1^T]x = 2x^T\mathbb{E}[\phi_0\phi_1^T]x. \tag{128}$$

From this follows $x^TMx \geq 0$ with equality only if $x$ is in the kernel of $\mathbb{E}[\phi_0\phi_0^T]$ and hence the claim. □

# G Symmetrized distribution is closer to uniform distribution

It is intuitive, that a reversible distribution of transitions is closer to the uniform distribution. The Kullback-Leibler divergence yields a particularly easy way to prove this intuition. Assume our state space $\mathcal{S}$ is a undirected graph $G = (\mathcal{S}, E)$, that is, we are in a deterministic setting, and if one can go from $s_1$ to $s_2$ then one can also go back. Then, together with the stationary distribution $\pi(s)$, the transition probabilities $p(s|s')$ define a joint probability on the set of edges (with orientation) of the graph, i.e.

$$p((s_1, s_2)) = p(s_2|s_1)\pi(s_1), \tag{129}$$

with $(s_1, s_2)$ denoting the directed edge from $s_1$ to $s_2$. That is, if we take the transition probabilities

$$tp(s_2|s_1) + (1-t)p(s_1|s_2)\frac{\pi(s_2)}{\pi(s_1)}, \tag{130}$$

then the joint distribution would be simply

$$tp(s_2|s_1)\pi(s_1) + (1-t)p(s_1|s_2)\frac{\pi(s_2)}{\cdot} \tag{131}$$

Now, if we were to observe transitions uniformly, then we have

$$p((s_1, s_2)) = \mathbb{I}[(s_1, s_2) \in E]\frac{1}{2|E|} =: q((s_1, s_2)), \tag{132}$$

where the factor 2 appears because we consider each undirected edge twice. Note that this is the joint probability that is obtained when one classically defines the random walk on an undirected graph with

$$p(s_2|s_1) = \mathbb{I}[(s_1, s_2) \in E]\frac{1}{deg(s_1)} \tag{133}$$

with $deg(s_1) = \sum_{s'} \mathbb{I}[(s_1, s') \in E]$ the degree. Indeed, weighting the above equation with the stationary distribution $\pi(s) = \frac{deg(s)}{2|E|}$ yields the uniform distribution over edges.

Now, the Kullback-Leibler divergence between any probability distribution and the uniform distribution is just

$$\mathrm{D_{KL}}(p||q) = \sum_{(s_1,s_2)} \ln p((s_1, s_1))p((s_1, s_2) + c, \tag{134}$$

with $c$ not depending on $c$ -i.e, it is just given by the negative entropy of $p$. Hence, the following lemma shows that indeed choosing $t = \frac{1}{2}$ in Equation 130 leads us closest to uniform.

**Lemma 7.** *Let $X \in \mathcal{X}$ be a random variable with density $p$, where $\mathcal{X}$ admits an involution, that is a bijective, measure-preserving map $\sigma : \mathcal{X} \to \mathcal{X}$ such that $\sigma^2 = Id$. Let $H(\cdot)$ be the entropy. Then for any $\frac{1}{2} \neq t \in [0, 1]$:*

$$H(\frac{1}{2}(p + p \circ \sigma)) \geq H(tp + (1-t)p \circ \sigma) \tag{135}$$

*and equality holds iff both densities are equal.*

*Proof.* By the strict concavity of entropy and the convexity of the set $\{tp + (1 - t)p \circ \sigma | t \in [0, 1]\}$, there is a unique maximum. Differentiating with respect to $t$ yields

$$\frac{d}{dt}H(tp + (1-t)p \circ \sigma) = \frac{d}{dt}\int_{\mathcal{X}} tp + (1-t)p \circ \sigma \ln tp + (1-t)p \circ \sigma d\mu \tag{136}$$

$$= 2\int_{\mathcal{X}} (p - p \circ \sigma) \ln tp + (1-t)p \circ \sigma d\mu \tag{137}$$

Now at $t = \frac{1}{2}$, the term $\ln \frac{1}{2}(p + p \circ \sigma)$ is invariant under $\sigma$, while the term $p - p \circ \sigma$ is skew-symmetric under $\sigma$. Hence, the integral vanishes. □

## H Proof that symmetrization of optimal policy is stable

Here, we want to show that an optimal policy remains optimal even if the value function is computed under symmetrized transition probabilities. Before we do so, we will however first state an useful identity for the successor representations.

**Definition 1.** *Under a fixed policy $\pi$, for $S_t$ a random walk with transition probabilities $P(s'|s) = \sum_a p(s'|a,s)\pi(a|s)$, we define the first hitting times*

$$\tau_{k+1}^s = \inf\{t > \tau_k^s \in \mathbb{N} | S_t = s\}, \tau_0^s = 0, \tag{138}$$

*where if the case that the set is empty we set the value to $\infty$. For a given pair of states $s, s'$ we define*

$$\mathcal{T}(s'|s) = \sup\{k \geq 0 | \mathbb{P}[\tau_k^{s'} < \infty | S_0 = s] > 0\}. \tag{139}$$

*Note that $\mathcal{T}(s'|s)$ only takes the values $\{0, 1, \infty\}$.*

It is easy to see that by, the Markovian property, the distribution of these stopping times does not change, in the sense that

$$\tau_{k+1}^s - \tau_k^s | \tau_k^s < \infty \sim \tau_1^s, \tag{140}$$

where for the left hand side we used the convention $\infty - c = \infty$ for any finite $c$. Using this, we have the following expression for the Successor representation in terms of stopping times:

**Proposition 2.** *For the successor representation $M = (Id - \gamma P)^{-1}$ we have*

$$M_{ss'} = \delta(s,s') + \begin{cases} 0, \mathcal{T}(s'|s) = 0 \\ \mathbb{E}[\gamma^{\tau_1^{s'}} | S_0 = s], \mathcal{T}(s'|s) = 1 \\ \frac{\mathbb{E}[\gamma^{\tau_1^{s'}}] | S_0 = s]}{1 - \mathbb{E}[\gamma^{\tau_1^{s'}}] | S_0 = s']}, \mathcal{T}(s'|s) = \infty \end{cases} \tag{141}$$

*Proof.* We note that

$$\begin{aligned} M_{ss'} &= (Id - \gamma P)^{-1}_{ss'} &(142)\\ &= \sum_{t=0}^{\infty} \gamma^t P^t_{ss'} &(143)\\ &= \sum_{t=0}^{\infty} \gamma^t \mathbb{P}[S_t = s' | S_0 = s] &(144)\\ &= \sum_{t=0}^{\infty} \gamma^t \sum_{k=0}^{\infty} \mathbb{P}[S_t = s', \tau_k^{s'} = t | S_0 = s] &(145)\\ &= \sum_{t=0}^{\infty} \gamma^t \sum_{k=0}^{\mathcal{T}(s'|s)} \mathbb{P}[S_t = s', \tau_k^{s'} = t | S_0 = s] &(146) \end{aligned}$$

where we have simply included a sum over all possible values for the hitting times and then included only the cases where those are finite.

Now note that in the above sum

$$\mathbb{P}[S_t = s', \tau_k^{s'} = t | S_0 = s] = \begin{cases} 1, k = t = 0, s = s' \\ \mathbb{P}[\tau_k^{s'} = t | S_0 = s], else. \end{cases} \tag{147}$$

Hence we get

$$= \delta(s, s') + \sum_{t=0}^{\infty} \gamma^t \sum_{k=1}^{\mathcal{T}(s'|s)} \mathbb{P}[\tau_k^{s'} = t \| S_0 = s] \tag{148}$$

$$= \delta(s, s') + \sum_{k=1}^{\mathcal{T}(s'|s)} \sum_{t=0}^{\infty} \gamma^t \mathbb{P}[\tau_k^{s'} = t | S_0 = s] \tag{149}$$

$$= \delta(s, s') + \sum_{k=1}^{\mathcal{T}(s'|s)} \mathbb{E}\left[\gamma^{\tau_k^{s'}} | S_0 = s\right]. \tag{150}$$

In particular, if $\mathcal{T}(s'|s) = 0$, we have $M(s'|s) = \delta(s, s')$. In the case that $\mathcal{T}(s'|s) = 1$, we get

$$M_{ss'} = \delta(s, s') + \mathbb{E}\left[\gamma^{\tau_1^{s'}} | S_0 = s\right]. \tag{151}$$

Now, for the remaining case $\mathcal{T}(s'|s) = \infty$ first note that

$$\gamma^{\tau_k^{s'}} = \prod_{l=0}^{k-1} \gamma^{\tau_{l+1}^{s'} - \tau_l^{s'}}, \tag{152}$$

and that for $l > 0$ we have $\tau_{l+1}^{s'} - \tau_l^{s'}$ is independent of $S_0$ and since at $\tau_l^{s'}$, $S_l = s'$, we have

$$\mathbb{E}\left[\tau_{l+1}^{s'} - \tau_l^{s'}\right] = \mathbb{E}\left[\tau_{l+1}^{s'} - \tau_l^{s'} | S_0 = s'\right] = \mathbb{E}\left[\tau_1^{s'} | S_0 = s'\right]. \tag{153}$$

Hence,

$$\mathbb{E}\left[\gamma^{\tau_k^{s'}} \| S_0 = s\right] = \mathbb{E}\left[\prod_{l=0}^{k-1} \gamma^{\tau_{l+1}^{s'} - \tau_l^{s'}} | S_0 = s\right] \tag{154}$$

$$= \mathbb{E}\left[\prod_{l=1}^{k-1} \gamma^{\tau_{l+1}^{s'} - \tau_l^{s'}}\right] \mathbb{E}\left[\gamma^{\tau_1^{s'} - \tau_0^{s'}} | S_0 = s\right] \tag{155}$$

$$= \mathbb{E}\left[\gamma^{\tau_1^{s'}} | S_0 = s'\right]^{(k-1)\delta_{k>1}} \mathbb{E}\left[\gamma^{\tau_1^{s'}} | S_0 = s\right]^{\delta_{k>0}}. \tag{156}$$

Plugging this into the sum formula yields

$$M_{ss'} = \delta(s,s') + \sum_{k=1}^{\infty} \mathbb{E}\left[\gamma^{\tau_k^{s'}} | S_0 = s\right] \tag{157}$$

$$\delta(s,s') + \mathbb{E}\left[\gamma^{\tau_1^{s'}} | S_0 = s\right] \left(\sum_{k=0}^{\infty} \mathbb{E}\left[\gamma^{\tau_1^{s'}} | S_0 = s'\right]^k\right) \tag{158}$$

$$= \delta(s,s') + \mathbb{E}\left[\gamma^{\tau_1^{s'}} | S_0 = s\right] \left(1 - \mathbb{E}\left[\gamma^{\tau_1^{s'}} | S_0 = s'\right]\right)^{-1} \tag{159}$$

$$\tag{160}$$

as claimed. □

**Corollary 2.** *In the setting with a unit reward $e_{s^*}$, the value function is completely determined by the expected $\gamma$ to the power of the first hitting time, that is*

$$V(s) \geq V(\tilde{s}) \iff \mathbb{E}[\gamma^{\tau_1^{s^*}} | S_0 = s] \geq \mathbb{E}[\gamma^{\tau_1^{s^*}} | S_0 = \tilde{s}]. \tag{161}$$

In particular, consider the deterministic setting - that is, the transitions $p(s'|a,s)$ are deterministic and hence the state-space becomes a directed graph, where a directed edge exists from state $s$ to state $s'$ if there is an action $a$ which (deterministically) leads from states $s$ to state $s'$. The task to navigate to state $s'$ is inherently finite/absorbing, but we can extend the time horizion to infinity by simply allowing an action that stays in the same state as one is (which hence is the optimal action once one is at the target state). In this deterministic setting, an optimal policy has to always decrease the distance to the target - hence, we have under an optimal policy, where the target is $s^*$,

$$(\tau_1^{s^*} | S_0 = s) = \delta(s \neq s^*) d(s,s^*) + \delta(s = s^*), \tag{162}$$

where $d(s,s')$ is the shortest number of steps necessary to get from $s$ to $s'$ (which is not necessarily symmetric). In particular, every non-target state $s'$ should never be returned to, and starting from state $s$, only states lying on shortest paths from $s$ to $s^*$ should be visited (where some of those shortest paths may still not be visited by the policy, as there might be multiple such paths). In summary:

**Corollary 3.** *In the directed graph setting navigating to target $s^*$, for an optimal policy $\pi^*$, the successor representation becomes*

$$M_{ss'} = \delta(s,s') + \begin{cases} 0, s = s' \neq s^* \\ 0, s \neq s', s' \textit{not on a shortest path from } s \textit{ to } s^* \textit{ which the policy visits} \\ \gamma^{d(s,s')}, s' \neq s^* \neq s \\ \frac{\gamma^{d(s,s')}}{1-\gamma^{d(s,s')}}, s' = s^* \end{cases} \tag{163}$$

Having understood this simple structure of the $SR$ under an optimal policy, we can also understand why a symmetrization does not impede solving the task. First, we have to slightly generalize our definition of the symmetrization of transition probabilities, as the one we used in our biological model only works in the case where the resulting Markov chain is ergodic. This is not the typical situation in navigation problems. Rather, in navigation tasks, under an optimal policy, one will navigate towards a goal on the shortest possible path and then typically the episode ends. Thus one could on the hand imagine an absorbing state at the target, but this then does not lend itself nicely to the generalization setting where the target should change but the environmental dynamics should stay the same. Instead, one could then not introduce an absorbing state in the environment transitions per se, and just include a self-transition at the target state - but still stop the episode accordingly when the reward is reached.

We thus would want to define the reverse process in this case as a process which always moves away from the target state $s^*$, until it reaches a state with maximal distance to the target, where it stays (or rather, the episode ends) - hence mirroring the behaviour of the optimal policies. That is formally, if $P$ are the transition probabilities under the optimal policy, and $d_{\max} = \max_s d(s, s^*)$

$$P^{reverse}_{s,s'} = \begin{cases} \delta_{s,s'}, & d(s,s^*) = d_{\max} \\ P(S_{\tau^s_1 - 1} = s' | \tau^s_1 < \infty), & d(s,s^*) < d_{\max} \end{cases} \tag{164}$$

The symmetrized process is then the one with transition probabilities given by

$$q = \frac{1}{2}(P + P_{reverse}). \tag{165}$$

The value function induced by $q$ for the navigational problem (i.e. reward vector $e_{s^*}$) is then

$$V^{s*}_q(s) = \sum_{k=0}^{\infty} \gamma^k q_k(s^*|s). \tag{166}$$

Recall that a policy $\pi$ is optimal for a value function $V$ if the policy always selects actions which maximize the value function. We have

**Proposition 3.** *Let $\pi$ be an optimal policy in the deterministic navigation problem with target state $s^*$ and let $q$ be the corresponding symmetrized process. Then $\pi$ is also an optimal policy for $V^{s*}_q$.*

*Proof.* First, let us define the sets

$$D_m = \{s \in \mathcal{S} | d(s,s^*) = m\}. \tag{167}$$

It is obvious that under the optimal policy, for the forward process, we have

$$p(S_t \in D_m | S_{t-1} \in D_{m+1}) = 1, m > 0 \tag{168}$$

and

$$p(S_{t-1} \in D_m | S_t \in D_{m-1}, \tau^{s^*}_1 > t) = 1, m < d_{\max} \tag{169}$$

This means that for the symmetrized process, with transition probabilities $q = \frac{1}{2}(P + P^{reverse})$, we actually have

$$q(D_{\min(m+1,\max(d(s,s*))}|D_m) = q(D_{\max(m-1,0)}|D_m) = \frac{1}{2}. \tag{170}$$

That is, the symmetrized process with probability 1/2 either increases or decreases the distance to the target state $s^*$ by 1, with exception at the boundaries (i.e. at maximal distance or zero distance), where it stays with probability $\frac{1}{2}$. Now consider the value-function under $q$, which depends on the sum of the $k-$step probabilities $q_k$. But the $k-$ step probabilities can be written as

$$q_k(s^*|s) = \sum_{(l_0,l_1,\dots,l_k)} q(D_0|D_{l_0})q(D_{l_i}|D_{l_{i+1}})\cdots q(D_{l_k}|s), \tag{171}$$

where the sum runs over all possible alignments such that one can reach $s^*$ after $k$ steps from $s$. This implies that the value-function can be completely determined by looking at the transition probabilities on the coarse-grained state space of level sets of the distance function, which corresponds to a line graph which self-loops at the ends. To be more precise, the value of $s$ under $q$ is determined simply by

$$V_q(s) = V(D_{d(s,s^*)}). \tag{172}$$

] This tells us that the value of the state is a function only of the distance to the target state. Now we only need to show that this function decreases strictly with distance. To do this, we can simply study the line-graph, or rather the process with transition matrix

$$Q = \begin{pmatrix} \frac{1}{2} & \frac{1}{2} & 0 & 0 & \dots \\ \frac{1}{2} & 0 & \frac{1}{2} & 0 & \dots \\ 0 & \frac{1}{2} & 0 & \frac{1}{2} & \dots \\ \dots & \dots & \dots & \dots & \dots \\ \dots & 0 & 0 & \frac{1}{2} & \frac{1}{2}. \end{pmatrix} \tag{173}$$

Now let $d(s, s*) < d(s', s*)$. It is then clear that $\mathbb{P}[\tau_1^{s^*} = t|S_0 = s] < \mathbb{P}[\tau_1^{s^*} = t|S_0 = s']$, since any path from $s'$ to $s^*$ has to go through $s$. Hence, recalling proposition 2, also

$$V_q(s) = M^q_{ss*} = \frac{\mathbb{E}[\gamma^{\tau_1^{s^*}}|S_0 = s]}{1 - \mathbb{E}[\gamma^{\tau_1^{s^*}}|S_0 = s^*]} < \frac{\mathbb{E}[\gamma^{\tau_1^{s^*}}|S_0 = s']}{1 - \mathbb{E}[\gamma^{\tau_1^{s^*}}|S_0 = s^*]} = V_q(s'). \tag{174}$$

This shows that the indeed the value-function decreases strictly with distance, and hence the optimal policy $\pi$ which we started with, also strictly decreases $V_q$ and hence also is an optimal policy for $q$, which concludes the proof. □

# References

[1] Sutton RS, Barto AG. Reinforcement learning: An introduction. MIT press; 2018.

[2] Fang C, Aronov D, Abbott L, Mackevicius EL. Neural learning rules for generating flexible predictions and computing the successor representation. Elife. 2023;12:e80680.

[3] Huang Y, Rao RP. Predictive coding. Wiley Interdisciplinary Reviews: Cognitive Science. 2011;2(5):580–593.

[4] Machado MC, Rosenbaum C, Guo X, Liu M, Tesauro G, Campbell M. Eigenoption discovery through the deep successor representation. arXiv preprint arXiv:171011089. 2017;.

[5] Machado MC, Bellemare MG, Bowling M. A laplacian framework for option discovery in reinforcement learning. In: International Conference on Machine Learning. PMLR; 2017. p. 2295–2304.

[6] Mahadevan S, Maggioni M. Proto-value Functions: A Laplacian Framework for Learning Representation and Control in Markov Decision Processes. Journal of Machine Learning Research. 2007;8(10).

[7] Stachenfeld KL, Botvinick M, Gershman SJ. Design principles of the hippocampal cognitive map. Advances in neural information processing systems. 2014;27.

[8] Stachenfeld KL, Botvinick MM, Gershman SJ. The hippocampus as a predictive map. Nature neuroscience. 2017;20(11):1643–1653.

[9] Chung FR. Laplacians of graphs and Cheeger's inequalities. Combinatorics, Paul Erdos is Eighty. 1996;2(157-172):13–2.

[10] Jost J, Mulas R. Cheeger-like inequalities for the largest eigenvalue of the graph Laplace Operator. arXiv preprint arXiv:191012233. 2019;.

[11] Von Luxburg U. A tutorial on spectral clustering. Statistics and computing. 2007;17:395–416.

[12] Sprekeler H. On the relation of slow feature analysis and laplacian eigenmaps. Neural computation. 2011;23(12):3287–3302.

[13] Chung F. Laplacians and the Cheeger inequality for directed graphs. Annals of Combinatorics. 2005;9:1–19.

[14] Johns J, Mahadevan S. Constructing basis functions from directed graphs for value function approximation. In: Proceedings of the 24th international conference on Machine learning; 2007. p. 385–392.

[15] Wu Y, Tucker G, Nachum O. The laplacian in rl: Learning representations with efficient approximations. arXiv preprint arXiv:181004586. 2018;.

[16] Dayan P, Sejnowski TJ. TD ($\lambda$) converges with probability 1. Machine Learning. 1994;14:295–301.

[17] Kushner HJ, Clark DS. Stochastic approximation methods for constrained and unconstrained systems. vol. 26. Springer Science & Business Media; 2012.
