# Supplementary material for: Impact of symmetry in local learning rules on predictive neural representations and generalization in spatial navigation
Source: PLoS Comput Biol. 2025 Jun 23;21(6):e1013056. doi: 10.1371/journal.pcbi.1013056 (PMC12184951; doi:10.1371/journal.pcbi.1013056)
Supplement: Attachment — Submitted filename: rebuttal_2.pdf [file pcbi.1013056.s008.pdf]

Dear Reviewer, thank you very much for the feedback of our manuscript and for going through all derivations and equations, this was very helpful. We will respond to your comments below:

There are two ways to read the paper. Either you discard the proofs (and thus this appendices) or you read everything. - If you discard the mathematical developments of the appendices, it is nearly impossible to follow the analysis. The equations are spread out in the entire documents (appendices) and not all notations, formulas, etc can be found. - If you choose to read the appendix and follow the mathematics thoroughly, then they are several gaps which prevent from having a complete understanding of the developments. This is mainly because, the equations are not properly spelt out and it is very difficult to follow some of the arguments (see below in minors revisions). It think it is better to be overly verbose (and repeat equations).

We do appreciate that indeed it was not as straightforward to read our first manuscript. We hope that with the changes we made, it will be easier to follow along now. However, we decided to keep the general outline, where the main text is mainly presenting results and further computations are deferred to methods section or appendices. Although the ones among us with a more mathematical background would generally be inclined to a more computation-heavy exposition, we want our paper to be also accessible to read for a more experimentally oriented audience - as those may draw inspiration from this work to test some of the predictions made here. We still agree that for the audience which wanted to follow all arguments, the manuscript needed to be more detailed. We thus worked through the manuscript again, and made things more explicit whenever it seemed adequate. In particular, we adrressed the points raised by you below.

- section 2.3. At this stage, most readers are lost. What is the dynamics for p_t (I guess 24-25), which weight W^f, W^r is affected by plasticity? We have included at this point in the manuscript now a full description of all the updates of activities and weights in one big equation, which reads

$$
\begin{aligned}
p^1(t+1) &= p^1(t) + \varepsilon_p \Delta p^1(p^1(t), W^r(t), \phi^1(S_t)) \quad (1)\\
p^2(t+1) &= p^2(t) + \varepsilon_p \Delta p^2(p^1(t), p^2(t), W^f(t), \phi^2(S_t))\\
W^r(t+1) &= W^r(t) + \varepsilon_W \Delta W^r(p^1(t), p^1(t+1), W^r(t), \alpha^r, \beta^r)\\
W^f(t+1) &= W^f(t) + \varepsilon_W \Delta W^f(p^1(t), p^1(t+1), p^2(t), p^2(t+1), W^f(t), \alpha^f, \beta^f)
\end{aligned}
$$

$$
\begin{aligned}
&\Delta p^1(p^1, W^r, \phi^1) = -p^1 + \sigma(\gamma_1 W^r p^1 + (1-\gamma_1)\phi^1) \qquad (2)\\
&\Delta p^2(p^1, p^2, W^f, \phi^2) = -p^2 + \sigma(\gamma_2 W^f p^1 + (1-\gamma_1)\phi^2)\\
&\Delta W^r(p^1, p^{1\prime}, W^r, \alpha^r, \beta^r) = \alpha^r(p^{1\prime} - W^r p^1)p^{1^T} + \beta^r(p^1 - W^r p^{1\prime})p^{1\prime^T}\\
&\Delta W^f(p^1, p^{1\prime}, p^2, p^{2\prime}, W^f, \alpha^f, \beta^f) = \alpha^f(p^{2\prime} - W^f p^1)p^{1^T} + \beta^r(p^2 - W^f p^{1\prime})p^{1\prime^T}
\end{aligned}
$$

Obviously, I have read everything. Appendix A is not really understandable. Appendix B is OK. Appendix D is kind of OK despite D.1 being not. The proof of th.1 is good. Appendix E is OK. Appendix F is good: it is the amount of details, precision that I would expect.

We went through all appendices again and made all computations more explicit and added detail where needed.

minor revision - in the abstract, change "center of mass" into "mean"?

Although we agree that this is essentially the mean, the experimental community seems to use the term center of mass when referring to place fields. Thus, to keep accessible to this community, we left it as is. We have however made explicit in the definition of centre of mass that it is essentially a mean.

- author summary "symmetric in time" -¿ "symmetric wrt to exchange of input timings"?

We changed it to your suggestion.

- page 3, you mention Bi and Poo for the CA3-CA1 synapse but Bi and Poo focused on cultured neurons

You are right, we should have been more careful there. The passage now reads *However, it has been suggested that different learning rules might be in place at the respective synapses: the Schaffer collateral- synapse to CA1 pyramidal cells is classically believed to obey the rules of STDP (**??**), which in its stereotypical form requires presynaptic increased activity to precede postsynaptic increased activity for an increase in strength of synaptic connection (**??**).*

- page 5, are you sure about (4)?

Thanks for pointing this out, this was indeed not formulated the way we intended to.

- page 5 what are λ in (5-6)?

We have discarded λ and are now using γ both for the parameter of the network and for the reinforcement learning parameter, and we also explain this double

use of notation as they are meant to be equivalent. We included this sentence

The $\gamma_i \in [0, 1]$ are scalar valued global gain factors which control the relative strengths of inputs to the populations. Note that we intentionally used the same symbol as for the timescale factors in the successor representation, as those will turn out to be equivalent.

- page 6: Figure 'C. I find very surprising the asymmetric learning rule case. You have the same input but DeltaW is completely different

Here it was not clear to us what you referred to. Indeed for the asymmetric learning rule, DeltaW should be different if we change the order of the inputs, as it is sensitive to which neuron is active first.

- page 21 I do not understand eq 36-37. Where is $\tilde{SF}_t(S)$ defined?

We included a sentence that makes clear that $\tilde{SF}_t$ is the current estimate of the true successor representation which the agent updates. It now reads: *Indeed, if $\tilde{SF}_t$ is the current estimate the agent has for the successor features, then consider update rules*

$$\tilde{SF}_{t+1}(S_t) = \tilde{SF}_t(S_t) + \varepsilon\Delta_t \tag{3}$$

$$\Delta_t = \phi(S_t) + \gamma\tilde{SF}_t(S_{t+1}) - \tilde{SF}_t(S_t). \tag{4}$$

Then we see that if $\tilde{SF}_t = SF_\phi$, we get $E[\Delta_t|S_t = s] = 0$. Thus, here $\phi(S_t) + \gamma\tilde{SF}_t(S_{t+1})$ is used as a bootstrapping estimate of the target $E[\phi(S_t)+\gamma\tilde{SF}_t(S_{t+1})|S_t = s]$, and $\tilde{SF}_t$ is updated according to the error to that target - in total, Δ_t is thus also called TD-error.

- page 22 above (40). Are you sure e_s is injective?

What needs to be injective is the map $s \mapsto e_s$, mapping a state to the corresponding unit vector. Since the dimensionality is assumed to be equal to the number of states, this is always injective.

- page 33, notation (64) is not very clear.

We assumed that you referred here to equation (104) and added additional explanation for that equation. It now reads
Now, we want to obtain the Jacobian from the expression above, that is we want to find a matrix that represents this derivative. To achieve this, we may vectorize the matrix U using the vectorization operator vec, which transforms a matrix into a vector by stacking the columns on top of each other. Using the well known identity

$$\text{vec}(ABC) = (C^T \otimes A)\,\text{vec}(B), \tag{5}$$

where $\otimes$ denotes the Kronecker product of matrices, we thus have ...

- Equation 66 is very difficult to find. Please make a lemma and

prove it.

We included a lemma that addresses this.

- theorem 1 page 37, what is alpha?

Good catch again, α was supposed to be ε.

- appendix D.1 What is Π? Please report the equations of the model fully. Π is a diagonal matrix with the values of the stationary distribution on the diagonal. We have added a reminder in that section.

- page 3, typo "temporal difference(TD)"
- page 33 type "retrieves A.More"
- prop 2 page 26. typo $(Id - \gamma P)^{-1})$. This typo can be found elsewhere
- type eq (185)

Thanks for spotting these!
